# Insights into Probiotic Prescription among Gastroenterologists and Other Healthcare Professionals: Evidence from an Italian Survey

**DOI:** 10.3390/jcm13164749

**Published:** 2024-08-13

**Authors:** Giovanni Marasco, Angelo Bruni, Olga Maria Nardone, Loris Riccardo Lopetuso

**Affiliations:** 1 Division of Internal Medicine and Digestive Physiopathology, IRCCS Azienda Ospedaliero-Universitaria di Bologna, 40138 Bologna, Italy; 2 Department of Medical and Surgical Sciences, University of Bologna, 40138 Bologna, Italy; angelo.bruni4@unibo.it; 3 Gastroenterology Unit, IRCCS Azienda Ospedaliero-Universitaria di Bologna, 40138 Bologna, Italy; 4 Gastroenterology, Department of Public Health, University Federico II of Naples, 80131 Naples, Italy; olgamaria.nardone@unina.it; 5 CEMAD—IBD UNIT—Unità Operativa Complessa di Medicina Interna e Gastroenterologia, Dipartimento di Scienze Gastroenterologiche, Endocrino-Metaboliche e Nefro-Urologiche, Fondazione Policlinico Universitario “A. Gemelli” IRCCS, 00168 Rome, Italy; lorisriccardo.lopetuso@policlinicogemelli.it

**Keywords:** probiotics, microbiota, survey

## Abstract

**Background**: Probiotics, which are live microorganisms that provide health benefits, have been extensively studied for their various clinical applications. However, despite their potential, high-quality data supporting their use in several gastrointestinal diseases are often lacking, and prescription behaviors can widely differ. This study aimed to assess different behaviors in probiotics knowledge and prescriptions among Italian gastroenterologists and healthcare professionals (HPs). **Methods**: A web-based electronic survey was distributed to all participants at the National Meeting of the Italian Young Gastroenterologist and Endoscopist Association (AGGEI) held in 2023. The survey investigated probiotic prescription practices for several gastrointestinal conditions, such as acute diarrhea, irritable bowel syndrome, inflammatory bowel disease, and diverticular disease. **Results**: Among 200 participants, 142 completed the survey, of whom 59 were gastroenterologists and 83 were HPs (surgeons, nutrition biologists, and other physicians). Significant differences were observed in the prescription of probiotics for the treatment of acute diarrhea and *H. pylori*. Both groups prescribed probiotics in monthly cycles for patients with IBS, although the majority prescribed multistrain formulations. Gastroenterologists were more likely to prescribe cyclic courses for IBS, while HPs tended to continue therapy by changing the probiotic strain in case of inefficacy. For ulcerative colitis, gastroenterologists prescribed probiotics more but for shorter durations. In Crohn’s disease, gastroenterologists prescribed probiotics less and were less likely to prescribe multistrain formulations. Regarding SUDD, gastroenterologists tended to prescribe probiotics less frequently, although without a significant difference, with similar rates of preference for multistrain formulations. **Conclusions**: This survey highlights heterogeneous behaviors in probiotic prescription between gastroenterologists and HPs, with gastroenterologists more aligned with guidelines and available scientific evidence. Hence, enhancing probiotic education among healthcare professionals and gastroenterologists is crucial. Further studies are needed to better understand probiotics’ role in gastrointestinal disorders through large-scale randomized controlled trials.

## 1. Introduction

The term “probiotic”, as defined by the FAO/WHO, encompasses “live microorganisms that, when administered appropriately, confer health benefits to the host” [1]. They should be distinguished from prebiotics, which, instead, are defined as a substrate that is selectively utilized by host microorganisms conferring a health benefit [2]. Probiotics are able to restore intestinal health by balancing the microbiota, aiding digestion, and boosting the immune system [3,4]. They have been extensively studied and found to have diverse clinical applications, ranging from preventing acute diarrhea to managing complex conditions like inflammatory bowel disease (IBD) and irritable bowel syndrome (IBS) [3,5,6]. Moreover, probiotics hold promise in enhancing immune responses, preventing conditions like necrotizing enterocolitis in preterm neonates, and mitigating nonalcoholic fatty liver disease [7,8]. Beyond gastrointestinal disorders, probiotics and prebiotics showed promising data for managing a wide range of conditions, including bacterial vaginosis, atopic dermatitis, oral health issues, upper respiratory tract infections, and neurological diseases [9,10,11]. Ongoing research is exploring their role in preventing manifestations of metabolic syndrome [12,13].

Probiotics primarily restore gut microbiota balance and modulate the immune system, enhancing digestion, nutrient absorption, and overall immune response. Additionally, they produce antimicrobial substances, strengthen the intestinal barrier, and exhibit anti-inflammatory effects, contributing to their clinical benefits in various gastrointestinal and nongastrointestinal conditions [14,15,16]. However, their effectiveness is highly specific to the strain and dosage administered. Despite their potential benefits, challenges arise due to variations in trial methodologies, different study outcomes, and probiotic strains tested, since there are different strain-specific effects of probiotics. Moreover, in several clinical settings, uncertainties are sustained by gaps in understanding microbial-host interactions. Therefore, to guide clinical decisions, specific recommendations based on graded evidence levels are crucial [1,17]. As an example, while most guidelines suggest a possible probiotic efficacy in IBS as a whole category [18,19], the American Gastroenterology Association (AGA) has not provided sufficient evidence to endorse probiotics for clinical use in IBS [18,20]. A recent meta-analysis examined 82 trials involving 10,332 patients undergoing probiotics for IBS, finding moderate certainty evidence supporting the benefit of Escherichia strains for global symptoms, while certainty was lower for *Lactobacillus* strains and *Lactobacillus plantarum* 299V [21]. Similarly, very low certainty existed regarding combination probiotics and Bacillus strains for abdominal bloating or distension. However, across 55 trials involving over 7000 patients, the relative risk of experiencing adverse events with probiotics was not significantly higher than placebo [21].

As for IBD, the European Crohn’s and Colitis (ECCO) guidelines acknowledge the limited evidence supporting the use of prebiotics, probiotics, or their combination in patients with Crohn’s disease (CD) or in individuals with CD post-surgery [22]. Despite uncertainties, there is optimism about specific probiotic strains’ potential in managing ulcerative colitis (UC) [23]. Moreover, multistrain probiotics containing *Lactobacillus* bacteria, *Streptococcus*, and *Bifidobacteria* may effectively induce and maintain remission in UC [22,24]. Despite some probiotics showed to be effective for mild to moderate UC cases, a 2020 Cochrane meta-analysis reported limited evidence supporting their ability to induce remission in such cases and no evidence supporting their effectiveness in severe disease [25]. The use of probiotics has also been proposed in diverticular disease (DD) to counteract bacterial overgrowth resulting from altered colonic motility, which disrupts the gut microbiota and mucosal barrier, leading to inflammation and disease progression [26]. While studies suggest that probiotics may alleviate symptoms, definitive evidence is lacking due to study variability. A systematic review showed that high-quality data on the efficacy of probiotics in diverticular disease are still scant [27]. However, the absence of high-quality data regarding probiotic effectiveness in several gastrointestinal diseases can lead to heterogeneous behavior in their prescription in real-life clinical practice, which sometimes may not be supported by scientific evidence. Therefore, we aimed to assess the behavior in probiotics prescription in a group of young Italian gastroenterologists, who are supposed to be more focused on the management of gastrointestinal diseases, compared with a control group of other physicians/healthcare professionals (HPs) participating in an Italian National meeting. Our results may also be useful for highlighting the need for updates in this field.

## 2. Materials and Methods

### 2.1. Study Design

A prospective web-based survey was designed to evaluate the current behaviors in probiotics use in clinical settings among young gastroenterologists, GI trainees, and nonmedical healthcare professionals. The survey’s development entailed a videoconference meeting led by a task force consisting of three AGGEI representatives. Following internal discussion, the final questionnaire gained unanimous approval from the AGGEI Steering Committee prior to deployment. The survey was conducted in accordance with the World Medical Association Declaration of Helsinki. Written informed consent was obtained from participants in the study.

### 2.2. Development and Content of the Questionnaire

The survey, conducted in Italian (translation available in Supplementary Material S1), aimed to gather information on the behavior in participant prescription of probiotics in their daily clinical practice. Participants were asked to specify their geographical area, professional title, and workplace. Subsequently, they were encouraged to detail the probiotic formulations and prescription timelines for several gastrointestinal diseases, across a total of 28 multiple-choice questions. The questionnaire was divided into five sections: demographic information (Section S1), infectious diarrhea and *Helicobacter pylori* (Section S2), irritable bowel syndrome (Section S3), inflammatory bowel disease (Section S4), and diverticular disease (Section S5). The complete survey questions and responses are provided in the tables below.

### 2.3. Distribution of Questionnaire and Collection of Data

The electronic version of the survey was distributed via e-mail to all the AGGEI members participating in the annual National Meeting held in Rome from 24 to 25 November 2023. The survey was further emailed as a follow-up to participants of the meeting for one additional month.

### 2.4. Statistical Analysis

Data are presented as counts and percentages for the categorical variables and mean and standard deviation (SD) for the continuous variables. Data were compared between gastroenterologists and other HP groups in order to shed light on different probiotic prescription behaviors. The categorical variables were compared using the Chi-squared or Fisher’s exact tests as appropriate. For multiple categorical variables, the Chi-squared test of independence was used. The continuous variables were compared using the *t*-test or the Kruskal–Wallis test as appropriate. The probability values were two-sided; a probability value of less than 0.05 was considered statistically significant. Statistical analysis was performed with STATA 17.0 (SE, Standard Edition, College Station, TX, USA: StataCorp LP).

## 3. Results

### 3.1. Demographics and Professional Data

Among 200 participants, 142 (71%) completed the survey, of whom 59 (41.5%) were gastroenterologists and 83 (58.4%) were HPs (surgeons and other physicians, nutritionists). We found a significant difference in gender distribution between the two groups (males 58.6% gastroenterologist vs. 43% HP, *p* = 0.003). The distribution across geographical regions (northwest, northeast, central, southern, and islands) of participants in the survey was similar between the two groups (*p* = 0.902). Significant differences were found considering the practice environment, since the majority of gastroenterologists (50.8%) worked in academic hospitals, whereas HPs are more likely to be employed in private hospitals (32.9%) or other settings (50.5%, *p* < 0.001). Table 1 details the demographic and professional data of participants in the survey.

### 3.2. Knowledge of Microbiota and Probiotics

The correct definition of a probiotic was provided by a similar rate of participants in both groups (gastroenterologist 67.8% vs. HP: 72.5%, *p* = 0.280). Gastroenterologists demonstrated a broader awareness of the different probiotic genera, with a higher percentage reporting knowledge of more than 10 genera compared with HPs (53.3% vs. 24.1%, *p* = 0.001), and also showed better knowledge in identifying strains that have not been previously investigated in clinical practice (*p* < 0.001) (Supplementary Table S1).

### 3.3. Management of Acute Diarrhea and H. pylori Eradication

For the treatment of acute diarrhea (Supplementary Table S2), gastroenterologists were less prone to prescribe probiotics (45.8% vs. 90.11% HP, *p* < 0.001). Moreover, in this setting, gastroenterologists preferred single-strain formulations (91% vs. 72.8%), particularly those containing *Lactobacilli* (52.27% vs. 20.4% HP). Although HPs preferred multistrain formulations (27.3%) in this setting, the single-strain formulation preferred was *Saccharomyces* (36.3%). When exploring multistrain formulations, both groups preferred *Lactobacilli* and *Bifidobacteria* combinations (*p* = 0.068). In terms of length of probiotics administration, the majority of participants in both groups maintained probiotics for some days after symptoms resolution (30.3% gastroenterologist vs. 48.8% HP), although gastroenterologist preferred longer durations (14 days 28.2% vs. 21.5% HP, *p* = 0.013).

**Figure 1:**
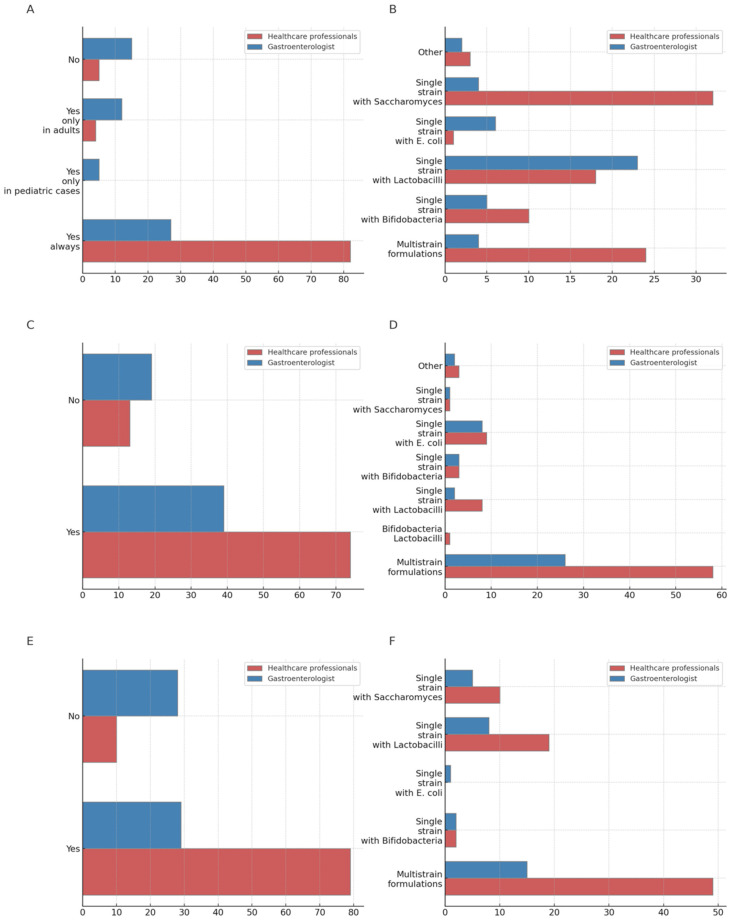
Use and prescription of probiotics for acute diarrhea, antibiotic-associated diarrhea, and *C. difficile* infection and treatment of *H. pylori* eradication (percentage): (**A**) Use of probiotics for acute diarrhea, (**B**) type of probiotic prescribed for acute Diarrhea, (**C**) use of probiotics to prevent antibiotic-associated diarrhea and *C. difficile* infection, (**D**) type of probiotic prescribed to prevent antibiotic-associated diarrhea and *C. difficile* infection, (**E**) use of probiotics as adjuncts in the treatment of *H. pylori* eradication, (**F**) type of probiotic prescribed as adjuncts in the treatment of *H. pylori* eradication.

Significant differences were observed between groups when asked about the use of probiotics for preventing antibiotic-associated diarrhea and *C. difficile* infection (gastroenterologists 67.2% vs. 85% HP, *p* = 0.011). For this purpose, both groups preferred multistrain formulations, although gastroenterologists preferred products with *Lactobacilli* and *Bifidobacteria*, while HPs preferred *Saccharomyces* and *Bifidobacteria*. Most of both groups prescribed probiotics during and after antibiotic therapy (gastroenterologists 73.8% vs. HPs 67.8%). As for *H. pylori* eradication therapy, a significantly lower rate of gastroenterologists prescribed probiotics in this setting (50.8% vs. HPs 88.7%, *p* < 0.001). However, a higher rate of gastroenterologists prescribed probiotics to reduce adverse events of antibiotic-eradicating regimens (30.5% vs. HPs 11.9%, *p* = 0.002) compared with HPs, rather than using probiotics for increasing eradication rates. Multistrain formulations were preferred by both groups in this setting (gastroenterologists 48.3% vs. HPs 61.2%) (Figure 1).

### 3.4. Irritable Bowel Syndrome

A significant rate of both subgroups (77.2% of gastroenterologists and 74.7% of HP) prescribed probiotics in monthly cycles for patients with IBS, although a significantly higher rate of gastroenterologists were not likely to prescribe probiotics in this setting (17.5% vs. 6.9% HP, *p* = 0.018). In terms of the type of probiotic prescribed, multistrain formulations were the preferred choice for both groups (55.3% gastroenterologist vs. 71.2% HP). As for single-strain formulations, gastroenterologists were more likely to prescribe *Lactobacilli* (25.5%), while HPs leaned toward *Bifidobacteria* (18.8%, *p* = 0.043). Among those using multistrain formulations, *Lactobacilli* were preferred by gastroenterologists (46.3%), while *Bifidobacteria* were preferred by HPs (49.3%). When using probiotics continuously for IBS, most gastroenterologists discontinued therapy after 2 months in case of inefficacy (52.7%), while HPs were more likely to change probiotic formulation (70%, *p* < 0.001).

**Figure 2:**
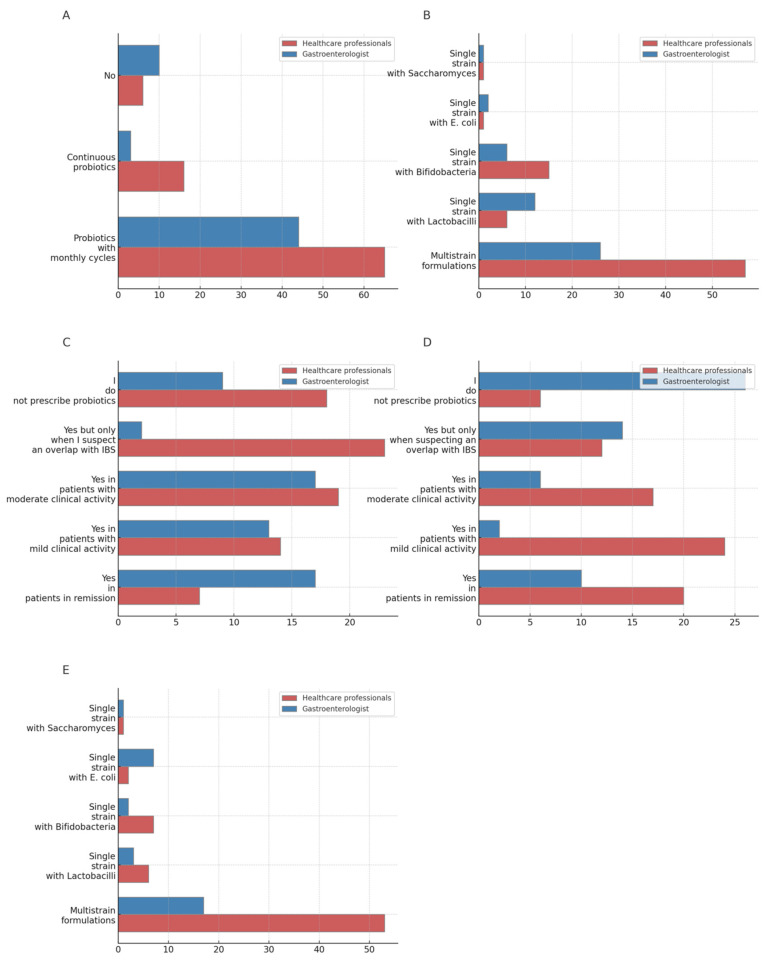
Prescription of probiotics for IBS and IBD (percentage): (**A**) Do you prescribe probiotics for patients with IBS? (**B**) What type of probiotic do you prescribe for IBS? (**C**) Do you prescribe probiotics for patients with Ulcerative Colitis? (**D**) Do you prescribe probiotics for patients with Crohn’s Disease? (**E**) What type of probiotic do you prescribe for ulcerative colitis and Crohn’s disease?

As for the cyclic probiotic administration schedule, the most common prescription duration was 10-14 days or more in both groups, without significant differences in total month duration (gastroenterologist 4.4 months vs. HPs 3.8) (Supplementary Table S3) (Figure 2).

### 3.5. Inflammatory Bowel Diseases

In the context of UC, gastroenterologists reported significantly higher rates of probiotic prescription across all disease activity stages compared with HPs (not prescribing 15.5% gastroenterologist vs. 22.2% HPs, *p* < 0.001), while, for CD, gastroenterologists exhibit significantly lower rates of probiotic prescription across all disease activity levels compared with other healthcare professionals (44.8% vs. 7.5% HP, *p* < 0.001). Multistrain formulations were the most preferred probiotics formulations in both groups for UC and CD, although when considering single-strain formulations, gastroenterologists were more likely to prescribe *E. coli* strains (23.3%) while HPs *Bifidobacteria* (10.1%).

As for the administration schedule for UC, in the case of continuous administration, gastroenterologists were more likely to discontinue therapy after 2 months in the case of inefficacy (38.7%), whereas HPs were more likely to change probiotic formulation (47.5%). Within the cyclic schedule of administration, gastroenterologists were more likely to prescribe 10-day cycles (44.1%), whereas HPs 14-day cycles (49.2%, *p* = 0.048), although without significant differences in total month duration.

Considering the continuous administration schedule in CD, gastroenterologists were more likely to suspend therapy after 2 months in case of inefficacy (57.6%), while HPs were more likely to change probiotic formulation (50%). As for cyclic administration in CD, no substantial differences were found between groups, with most of the respondents preferring 14-day cycles (gastroenterologists 44.4% vs. HPs 47%) without significant differences in total month duration (Supplementary Table S4) (Figure 2).

### 3.6. Diverticular Disease

**Figure 3:**
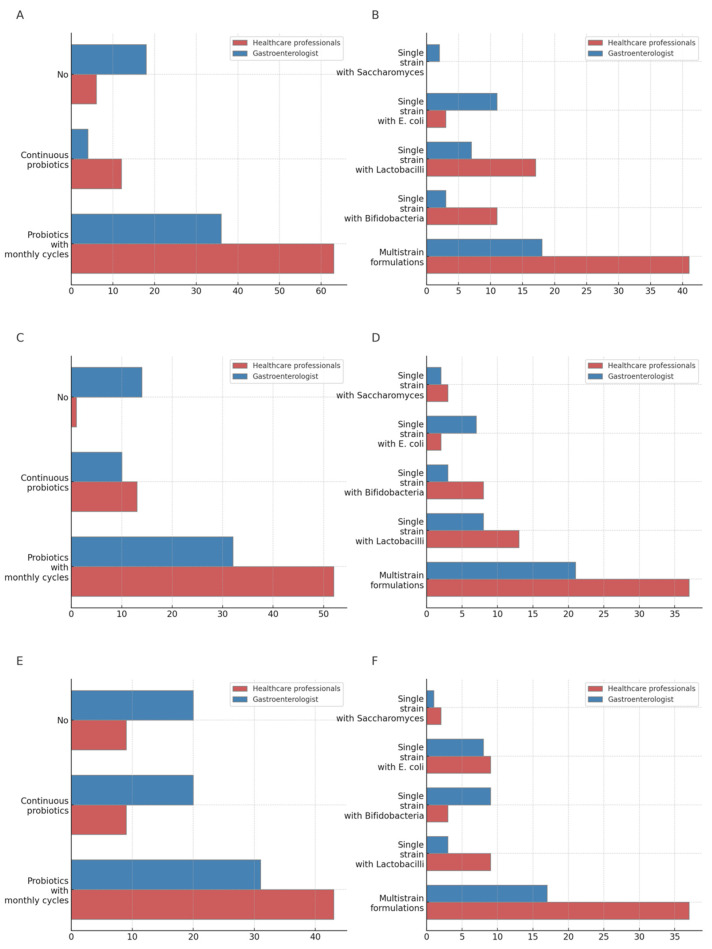
Prescription of probiotics for diverticulosis, SUDD, and acute diverticulitis (percentage): (**A**) In patients with a history of diverticulosis do you prescribe probiotics? (**B**) What type of probiotic do you prescribe for patients with diverticulosis? (**C**) Do you prescribe probiotics for patients with SUDD? (**D**) What type of probiotic do you prescribe for SUDD? (**E**) In patients with a history of acute diverticulitis, do you prescribe probiotics? (**F**) What type of probiotic do you prescribe in patients with a history of acute diverticulitis?

Gastroenterologists were less likely to prescribe probiotics in diverticulosis (31.1% vs. 7.4% HPs, *p* = 0.001). The majority in both groups preferred multistrains formulations (gastroenterologists 43.9% vs. HPs 56.9%), although when considering single-strain formulations, gastroenterologists were more likely to prescribe *E. coli* (26.8%) while HPs *Lactobacilli* (23.6%, *p* = 0.002). No significant differences were found in therapy duration in this setting. Also, for SUDD, gastroenterologists were less likely to prescribe probiotics (not prescribing 25% gastroenterologist vs. 13.3% HPs). The majority in both groups preferred multistrain formulations (gastroenterologists 51.2% vs. HPs 58.7%), and when considering single-strain formulations, both groups were more likely to prescribe *Lactobacilli* (gastroenterologists 19.5% vs. HPs 20.6%). No differences were found for the management of continuous probiotic administration in this setting, while looking at the cyclic administration schedule, the majority of gastroenterologists used 7-, 10-, or 14-day cycles (*p* = 0.013), while HPs were more prone to longer durations. No difference was found in the number of months suggested for cyclic administration. In acute diverticulitis, gastroenterologists were confirmed to be less prone to probiotics prescription (not prescribing 36.3% vs. HPs 13.2%, *p* = 0.002), without significant differences between groups regarding the probiotic formulation, although both groups preferred multistrain formulations. When probiotics were continuously used in this setting, gastroenterologists de-prescribed after 2 months of inefficacy, while HPs tended to continue this therapy, changing probiotic formulations. As for cyclic administration, the majority of participants in both groups prescribed cycles of 10 or more days with a trend in longer durations for HPs (*p* = 0.047), although gastroenterologists were more likely to prescribe probiotic cycles for more months (mean 5.7 months vs. 4.3 months HP, *p* = 0.025) (Supplementary Table S5) (Figure 3).

## 4. Discussion

This survey explored probiotic prescription behaviors among healthcare professionals compared with gastroenterologists and gastroenterology residents.

We found differences regarding gut microbiota general knowledge, its modulation in gastrointestinal diseases with probiotics, and duration of therapy. Particularly, gastroenterologists exhibited a more extensive knowledge of the probiotic genera currently available for administration.

Differences in prescription practices for acute diarrhea and antibiotic-associated diarrhea were found. Gastroenterologists showed a lower inclination to prescribe probiotics for acute diarrhea, but when prescribing, they preferred single-strain formulations and longer treatment durations. In contrast, HPs were more likely to prescribe and favor multistrain probiotics and shorter courses. However, there is currently limited evidence on the effectiveness of probiotics in treating acute infectious diarrhea in adults. A recent meta-analysis by Collinson et al. indicates that probiotics likely have little to no impact on the number of people who have diarrhea lasting 48 h or more, and it remains uncertain whether probiotics reduce the overall duration of diarrhea [28].

In addition, the American College of Gastroenterology (ACG) Clinical Guidelines on the management of acute diarrheal infections in adults [29] recommend against the routine use of probiotics for treating acute diarrhea in adults, except in cases of post-antibiotic-associated diarrhea, due to insufficient evidence of their effectiveness [18]. This suggests that the prescription behavior in this setting by HPs is not strongly supported by the existing evidence and guidelines [15,30].

This survey also describes similar probiotic prescriptions for IBS [18,31]. However, differences in prescribing schedules and discontinuation strategies were observed, highlighting divergent approaches to the management of IBS [18,19,32].

Notably, gastroenterologists leaned toward the prescription of multistrain formulations mainly containing *Lactobacilli* in monthly 10-day cycles. HPs also preferred multistrain formulations but containing *Bifidobacteria* and tended to modify the type of probiotic rather than discontinuing the therapy. Guidelines suggest the use of probiotics as a category; however, only the British Society of Gastroenterology (BSG) guidelines, as a consensus statement, suggest the continuous use of probiotics for 2 months and then reassessment of their efficacy [5]. As suggested by the meta-analysis by Goodoory et al., although with low levels of evidence, Lactobacilli prescribed by gastroenterologists may improve global symptoms, while Bifidobacteria may be more effective in reducing the perception of pain [21]. Therefore, HP prescription behavior in the setting of IBS, although not supported by scientific evidence, is in line with BSG guidelines suggestions.

Regarding IBD, we observed a distinct approach to the use of probiotics for UC and CD. The higher rate of probiotic prescriptions among gastroenterologists for UC is consistent with the consolidated evidence supporting the use of probiotics in UC management. Gastroenterologists prefer single-strain *E. coli* probiotics [33], with shorter cycles for UC and discontinuing ineffective therapy after a few months, while they are cautious in probiotics prescription in CD due to limited evidence.

Based on ECCO guidelines, probiotics can be useful alternatives to conventional medical therapy in UC and in treatment-resistant pouchitis as maintenance therapy after induction of remission with antibiotics [34,35]. More recently, a Cochrane meta-analysis supported the ability of probiotics to induce remission in mild-to-moderate UC cases without increasing the risk of treatment-related adverse events but with no evidence for their effectiveness in severe disease [25,36]. With regards to CD, limited evidence supporting the use of prebiotics or probiotics either in the induction or the maintenance of remission is available [36,37]. This may explain the lower rates of probiotic prescription for CD by gastroenterologists, reflecting uncertainties about their efficacy in this setting. On the other hand, HPs tended to overprescribe probiotics in IBD, especially in Crohn’s disease, where their use is not supported. However, future randomized clinical trials are required to validate the efficacy and safety of diverse probiotic multistrains in IBD.

Gastroenterologists were less likely to prescribe probiotics for diverticulosis compared with HPs. When they did prescribe, both groups preferred multistrain formulations, with gastroenterologists favoring *E. coli* strains and HPs favoring *Lactobacilli*. This trend in avoiding prescription in this setting remarked in the gastroenterologist group is supported by the current guidelines, which indicate limited evidence for the efficacy of probiotics in diverticulosis [25,38].

For SUDD, we found that both groups were more likely to prescribe probiotics in monthly cycles, but overall, gastroenterologists were also less likely to prescribe probiotics compared with HPs. Both groups preferred multistrain formulations, but when prescribing single-strain formulations, *Lactobacilli* were preferred. These behavior practices suggest that both groups believe in the potential benefit of probiotic use for the management of SUDD symptoms, although the overall quality of evidence of evidence supporting their use remains low [27,39]. Our results are also in line with a recently published wide prospective Italian cohort reporting probiotics use in 21–25% of SUDD patients [40].

In the case of acute diverticulitis, most of both groups prescribed probiotics, although gastroenterologists were less likely to prescribe probiotics continuously. When prescribed, both groups preferred multistrain formulations. Gastroenterologists tended to de-prescribe after two months if the therapy was ineffective, whereas HPs were more likely to continue the therapy by changing formulations. This practice is in contrast with guideline suggestions, since there is still limited evidence for their efficacy in preventing or managing acute diverticulitis [41,42]. Overall, gastroenterologists’ conservative approach in not prescribing probiotics in diverticular diseases and with an early withdrawal when prescribed is in line with the limited and heterogeneous evidence available in current guidelines [43]. The limitations of this study include a possible sampling bias of respondents which may have skewed results, as individuals who were not familiar with probiotics may not have returned the survey. Also, most gastroenterologists worked in academic hospitals, introducing a potential bias regarding real-life practices. In addition, the HP group was rather heterogeneous, since it included nongastroenterologist physicians such as surgeons and general practitioners, as well as other healthcare professionals such as dietitians and nutritionists. Moreover, the relatively limited number of respondents might have constrained the identification or influenced the observed differences. Lastly, even if we explored the use of probiotics in young gastroenterologists and other HPs in the specific setting of gastrointestinal diseases, our respondents may not be representative of all probiotic prescribers.

At the same time, this survey has several strengths. It provides a snapshot of the current knowledge and prescribing practices of probiotics among a group of young gastroenterologists and healthcare professionals in various disease settings. Moreover, we provided a commentary on our results in light of the most updated guidelines and scientific evidence. Finally, we also uncover probiotics prescription heterogeneity among gastroenterologists. Our results overall show a greater alignment of gastroenterologists with available evidence and guidelines in each setting compared with HPs. On one hand, this is not surprising, since most of these diseases require a focused evaluation made by a specialized physician, especially for IBD. On the other hand, most first-line therapies, especially within antibiotic-associated diarrhea, IBS, and diverticular disease fields, are often prescribed by HPs. This evidence underscores the importance of standardizing knowledge and the need for updates, especially for HPs, in the management of these gastrointestinal diseases, other than building networks for the optimization of the management of these patients. Probiotic overprescription may also prolong the therapeutic management of patients when used outside guidelines and clinical evidence, other than leading to patient dissatisfaction when they expect a clinical effect, finally further impacting patients’ quality of life.

In conclusion, probiotics are widely but heterogeneously employed in several gastroenterological settings. Gastroenterologists showed a more conscious approach to probiotic prescription, in line with available scientific evidence and clinical guidelines. However, in most settings explored, probiotic prescription heterogeneity is dependent on a lack of high-quality evidence. Therefore, academies and scientific societies should fill in this knowledge gap between gastroenterologists and other HPs, improving education on probiotic prescription. Moreover, further new high-quality well-conducted randomized controlled trials to support probiotic use in these settings are needed.

## Figures and Tables

**Table 1:** Demographic data of gastroenterologists and healthcare professionals who accepted to participate in the survey.

	Healthcare Professionals *n* (%) *n* = 83	Gastroenterologist *n* (%) *n* = 59	Total *n* (%) *n* =142	*p*-Value
**Age mean**	51.1 (13.7)	41.11 (15.6)	47.3 (15.2)	0.001
**Gender (Male)**	31 (34.0)	34 (58.6)	65 (43.0)	0.003
**Workplace Location**				0.902
Northwest	26 (28.5)	14 (23.7)	40 (26.6)	
Northeast	18 (19.7)	13 (22.0)	31 (20.6)	
Center	29 (31.8)	21 (35.5)	50 (33.3)	
South and Islands	18 (19.7)	11 (18.6)	29 (19.3)	
**Practice environment**				<0.001
University Hospital	7 (7.6)	30 (50.8)	37 (24.6)	
Nonuniversity Hospital	8 (8.7)	17 (28.8)	25 (16.6)	
Private Hospital	30 (32.9)	8 (13.5)	38 (25.3)	
Other	46 (50.5)	4 (6.7)	50 (33.3)	
**Clinical role**				<0.001
Gastroenterology Resident	0 (0.0)	23 (38.9)	23 (16.2)	
PhD Researcher	3 (3.61)	0 (0.0)	3 (2.1)	
Gastroenterologist	0 (0.0)	36 (61.0)	36 (25.3)	
Surgeon	8 (9.6)	0 (0.0)	8 (5.63)	
Nutrition Biologist	25 (30.1)	0 (0.0)	25 (17.6)	
Other	30 (3)	0 (0.0)	30 (21.1)	
Other Physician	17 (20.4)	0 (0.0)	17 (11.9)	

## Data Availability

The original contributions presented in the study are included in the article/Supplementary Materials, further inquiries can be directed to the corresponding author/s.

## Supplementary Materials

The following supporting information can be downloaded at https://www.mdpi.com/article/10.3390/jcm13164749/s1, Supplementary Material S1. Complete questionnaire used for the survey: Insights into probiotic prescription among gastroenterologists and other healthcare professionals; Table S1. Knowledge regarding microbiota and probiotics of gastroenterologists and healthcare professionals accepting to participate in the survey; Table S2. Behavior of gastroenterologists and healthcare professionals regarding the management of acute diarrhea and antibiotic-associated diarrhea with probiotics among participants in the survey; Table S3. Behavior of gastroenterologists and healthcare professionals regarding the management of irritable bowel syndrome with probiotics among participants in the survey; Table S4. Behavior of gastroenterologists and healthcare professionals regarding the management of inflammatory bowel disease with probiotics among participants in the survey; Table S5. Behavior of gastroenterologists and healthcare professionals regarding the management of diverticular disease with probiotics among participants in the survey.
