# Supplementary material for: Insights into Probiotic Prescription among Gastroenterologists and Other Healthcare Professionals: Evidence from an Italian Survey
Source: J Clin Med. 2024 Aug 13;13(16):4749. doi: 10.3390/jcm13164749 (PMC11355817; doi:10.3390/jcm13164749)
Supplement: Supplementary file 1 [file jcm-13-04749-s001.zip › jcm-3116935-supplementary.pdf]

Supplementary material S1. Complete questionnaire used for the survey: Insights into probiotic prescription among gastroenterologists and other healthcare professionals.

S1. Demographic and professional data

Select your gender:

- Male
- Female

Age_____________

Workplace Location:

- Northwest (Valle d'Aosta, Piedmont, Lombardy, Liguria)
- Northeast (Friuli-Venezia Giulia, Veneto, Emilia Romagna, Province of Trento/Bolzano)
- Central (Tuscany, Marche, Umbria, Lazio)
- South and Islands (Abruzzo, Molise, Campania, Apulia, Basilicata, Calabria, Sicily, Sardinia)

Where is your practice environment?

- University Hospital
- Non-university Hospital
- Private Hospital
- Other

What is your clinical role?

- Gastroenterology Resident
- PhD Researcher
- Gastroenterologist
- Surgeon
- Nutrition Biologist
- Dietitian
- Other physician
- Other

Knowledge regarding microbiota and probiotics

What is the correct definition of a probiotic?

- Different bacterial strains attributed with potential protective effects for the host organism.
- Live microorganisms that, when administered in adequate amounts, confer a health benefit to the host.
- Preparation of inanimate microorganisms and/or their components that confer a health benefit to the host.
- A selectively fermented ingredient that induces specific changes in the composition and/or activity of the gastrointestinal microbiota, thus conferring health benefits to the host.

How many genera (Lactobacillus spp, Bifidobacteria spp, Bacillus spp, etc.) of probiotics do you know?

- More than 5
- More than 7
- More than 9
- More than 10

Which of these strains has NOT been used as a probiotic in currently available clinical studies?

- Bacillus coagulans
- Enterococcus faecium
- Enterococcus casseliflavus
- Streptococcus salivarius

S2. Acute diarrhea and antibiotics use

Do you use probiotics for the treatment of acute diarrhea?

- Yes, always
- Yes, only in pediatric cases
- Yes, only in adults
- No

If you use probiotics for the treatment of acute diarrhea, what type of probiotic do you prescribe?

- Multistrain formulations
- Single strain with Lactobacilli
- Single strain with Bifidobacteria
- Single strain with E. coli
- Single strain with Saccharomyces
- Other________________________

If you use multistrain products for this indication, which bacterial genera do you prefer in the product? (multiple choice)

- Lactobacilli
- Bifidobacteria
- E. coli
- Saccharomyces
- Bacillus
- Streptococcus
- Enterococcus
- Pediococcus
- Lactococcus
- Clostridium

If you use probiotics for the treatment of acute diarrhea, for how many days do you prescribe the formulation?

- 5
- 7
- 10
- 14
- Until resolution of symptoms
- Maintaining therapy for some days after resolution of symptoms

Do you use probiotics to prevent antibiotic-associated diarrhea and C. difficile infection?

- Yes
- No

If you use probiotics to prevent antibiotic-associated diarrhea and C. difficile infection, what type of probiotic do you prescribe?

- Multistrain formulations
- Single strain with Lactobacilli
- Single strain with Bifidobacteria
- Single strain with E. coli
- Single strain with Saccharomyces
- Other________________________

If you use multistrain products for this indication, which bacterial genera do you prefer in the product? (multiple choice)

- Lactobacilli
- Bifidobacteria
- E. coli
- Saccharomyces

- Bacillus
- Streptococcus
- Enterococcus
- Pediococcus
- Lactococcus
- Clostridium

If you use probiotics to prevent antibiotic-associated diarrhea and C. difficile infection, what is your prescription schedule?

- Before starting antibiotic therapy
- Before and during antibiotic therapy
- Only during antibiotic therapy
- During and after antibiotic therapy

Do you use probiotics as adjuncts in the treatment of H. pylori eradication?

- Yes
- No

If you use probiotics in H. pylori eradication, how do you use them?

- As a single eradication therapy
- Together with antibiotic therapy to exploit the effect of probiotics in inhibiting H. pylori growth through spatial competition, utilization of energy substrates, and production of bacteriocins
- To reduce adverse events associated with antibiotic therapy to improve tolerability
- None of the above reasons

If you use probiotics in H. pylori eradication, what type of probiotic do you prescribe?

- Multistrain formulations
- Single strain with Lactobacilli
- Single strain with Bifidobacteria
- Single strain with E. coli
- Single strain with Saccharomyces
- Other________________________

S3. Irritable bowel syndrome

Do you prescribe probiotics for patients with IBS?

- Probiotics with monthly cycles
- Continuous probiotics
- No

What type of probiotic do you prescribe for IBS?

- Multistrain formulations
- Single strain with Lactobacilli
- Single strain with Bifidobacteria
- Single strain with E. coli
- Single strain with Saccharomyces
- Other________________________

If you use multistrain products for this indication, which bacterial genera do you prefer in the product? (multiple choice)

- Lactobacilli
- Bifidobacteria
- E. coli
- Saccharomyces
- Bacillus
- Streptococcus

- Enterococcus
- Pediococcus
- Lactococcus
- Clostridium

If you use probiotics continuously for IBS, when do you discontinue therapy?

- In case of inefficacy after 2 weeks
- In case of inefficacy after 4 weeks
- In case of inefficacy after 2 months
- I do not discontinue therapy but change the type of probiotic
- I still discontinue therapy after (indicate number) _____ weeks

If you use cyclic probiotics for IBS, for how many days do you prescribe the formulation?

- 5
- 7
- 10
- 14
- >14

If yes, for how many months? ______ (number of months)

S4. Inflammatory Bowel Diseases

Do you prescribe probiotics for patients with Ulcerative Colitis?

- Yes, in patients in remission
- Yes, in patients with mild clinical activity
- Yes, in patients with moderate clinical activity
- Yes, but only when I suspect an overlap with IBS
- I do not prescribe probiotics

Do you prescribe probiotics for patients with Crohn's Disease?

- Yes, in patients in remission
- Yes, in patients with mild clinical activity
- Yes, in patients with moderate clinical activity
- Yes, but only when suspecting an overlap with IBS
- I do not prescribe probiotics

What type of probiotic do you prescribe for Ulcerative Colitis and Crohn's Disease?

- Multistrain formulations
- Single strain with Lactobacilli
- Single strain with Bifidobacteria
- Single strain with E. coli
- Single strain with Saccharomyces
- Other________________________

If you use continuous probiotics for Ulcerative Colitis, when do you discontinue therapy?

- In case of inefficacy after 2 weeks
- In case of inefficacy after 4 weeks
- In case of inefficacy after 2 months
- I do not discontinue therapy but but change the type of probiotic

If you use cyclic probiotics in Ulcerative Colitis, for how many days do you prescribe the formulation?

- 5
- 7
- 10

- 14
- >14

If yes, for how many months? ______ (number of months)

If you use continuous probiotics for Crohn's Disease, when do you suspend therapy?

- In case of inefficacy after 2 weeks
- In case of inefficacy after 4 weeks
- In case of inefficacy after 2 months
- I do not suspend therapy but change the type of probiotic

If you use cyclic probiotics for Crohn's Disease, for how many days do you prescribe the formulation?

- 5
- 7
- 10
- 14
- >14

If yes, for how many months? ______ (number of months)

S5. Diverticular Disease

In patients with a history of diverticulosis, do you prescribe probiotics?

- Probiotics with monthly cycles
- Continuous probiotics
- No

What type of probiotic do you prescribe for patients with a history of diverticulosis?

- Multistrain formulations
- Single strain with Lactobacilli
- Single strain with Bifidobacteria
- Single strain with E. coli
- Single strain with Saccharomyces
- Other________________________

If you use continuous probiotics for diverticulosis, when do you suspend therapy?

- In case of inefficacy after 2 weeks
- In case of inefficacy after 4 weeks
- In case of inefficacy after 2 months
- I do not suspend therapy but change the type of probiotic
- I suspend therapy after (indicate number) _____ weeks

If you use cyclic probiotics for diverticulosis, for how many days do you prescribe the formulation?

- 5
- 7
- 10
- 14
- Other_________

If yes, for how many months? ______ (number of months)

Do you prescribe probiotics for patients with SUDD (Symptomatic Uncomplicated Diverticular Disease)?

- Probiotics with monthly cycles
- Continuous probiotics

- No

What type of probiotic do you prescribe for SUDD?

- Multistrain formulations
- Single strain with Lactobacilli
- Single strain with Bifidobacteria
- Single strain with E. coli
- Single strain with Saccharomyces
- Other________________________

If you use continuous probiotics for SUDD, when do you suspend therapy?

- In case of inefficacy after 2 weeks
- In case of inefficacy after 4 weeks
- In case of inefficacy after 2 months
- I do not suspend therapy but change the type of probiotic

If you use cyclic probiotics for SUDD, for how many days do you prescribe the formulation?

- 5
- 7
- 10
- 14
- Other_________

If yes, for how many months? ______ (number of months)

If you use multistrain products for this indication, which bacterial genera do you prefer in the product? (multiple selection)

- Lactobacilli
- Bifidobacteria
- E. coli
- Saccharomyces
- Bacillus
- Streptococcus
- Enterococcus
- Pediococcus
- Lactococcus
- Clostridium

If you use probiotics continuously in SUDD, when do you discontinue the therapy?

- In case of inefficacy after 2 weeks
- In case of inefficacy after 4 weeks
- In case of inefficacy after 2 months
- I do not discontinue the therapy but change the type of probiotic
- I still discontinue the therapy after (indicate number) _____ weeks

If you use cyclical probiotics in SUDD, for how many days do you prescribe the formulation?

- 5
- 7
- 10
- 14
- Other_________
- If yes, for how many months? ______ (number of months)

In patients with a history of acute diverticulitis, do you prescribe probiotics?

- Probiotics with monthly cycles

- Continuous probiotics
- No

What type of probiotic do you prescribe in patients with a history of acute diverticulitis?

- Multistrain formulations
- Single strain with Lactobacilli
- Single strain with Bifidobacteria
- Single strain with E. coli
- Single strain with Saccharomyces
- Other________________________

If you use probiotics continuously in patients with a history of acute diverticulitis, when do you discontinue the therapy?

- In case of inefficacy after 2 weeks
- In case of inefficacy after 4 weeks
- In case of inefficacy after 2 months
- I do not discontinue the therapy but change the type of probiotic

If you use cyclical probiotics in patients with a history of acute diverticulitis, for how many days do you prescribe the formulation?

- 5
- 7
- 10
- 14
- Other_________

If yes, for how many months? ______ (number of months)

Supplementary Table S1. Knowledge regarding microbiota and probiotics of Gastroenterologists and Healthcare Professionals accepting to participate in the survey.

	**Healthcare professionals n (%) *n* = 83**	**Gastroenterologist n (%) *n* = 59**	**Total n (%) *n* =142**	***p*-Value**
**What is the correct definition of a probiotic?**				0.280
Different bacterial strains attributed with potential protective effects for the host organism.	13 (14.2)	12 (20.3)	25 (16.6)	
Live microorganisms that, when administered in adequate amounts, confer a health benefit to the host.	66 (72.5)	40 (67.8)	106 (70.6)	
Preparation of inanimate microorganisms and/or their components that confer a health benefit to the host.	4 (4.4)	0 (0.0)	4 (2.6)	
A selectively fermented ingredient that induces specific changes in the composition and/or activity of the gastrointestinal microbiota, thus conferring health benefits to the host.	8 (8.7)	7 (11.8)	15 (10.0)	
**Genera (Lactobacillus spp, Bifidobacteria spp, Bacillus spp, etc.) of probiotics known**				0.001
More than 5	29 (32.2)	29 (50.0)	58 (39.1)	
More than 7	10 (11.1)	15 (25.8)	25 (16.8)	
More than 9	3 (3.3)	0 (0.0)	3 (2.0)	
More than 10	48 (53.3)	14 (24.1)	62 (41.8)	
**Which of these strains has NOT been used as a probiotic in currently available clinical studies?**				<0.001
Bacillus coagulans	23 (26.7)	12 (21.0)	35 (24.8)	
Enterococcus faecium	8 (9.3)	20 (35.0)	28 (19.5)	
Enterococcus casseliflavus	47 (54.6)	14 (24.5)	61 (42.6)	
Streptococcus salivarius	8 (9.3)	11 (19.3)	19 (13.2)	

Supplementary Table S2. Behavior of Gastroenterologists and Healthcare Professionals regarding the management of acute diarrhea and antibiotic-associated diarrhea with probiotics among participants in the survey.

	**Healthcare professionals n (%) *n* = 83**	**Gastroenterologist n (%) *n* = 59**	**Total n (%) *n* =142**	***p*-Value**
**Use of probiotics for acute diarrhea**				<0.001
Yes, always	82 (90.1)	27 (45.7)	109 (72.6)	
Yes, only in pediatric cases	0 (0.0)	5 (8.4)	5 (3.3)	
Yes, only in adults	4 (4.4)	12 (20.3)	16 (10.6)	
No	5 (5.4)	15 (25.4)	20 (13.3)	
**Type of probiotics used for acute diarrhea**				<0.001
Multistrain formulations	24 (27.2)	4 (9.0)	28 (21.2)	
Single strain with Bifidobacteria	10 (11.3)	5 (11.3)	15 (11.3)	
Single strain with Lactobacilli	18 (20.4)	23 (52.2)	41 (31.0)	
Single strain with E. coli	1 (1.1)	6 (13.6)	7 (5.3)	
Single strain with Saccharomyces	32 (36.3)	4 (9.0)	36 (27.2)	
Other	3 (3.4)	2 (4.5)	5 (3.7)	
**Bacterial genera preferred in multistrains products for acute diarrhea**				0.068
Bifidobacteria	37 (41.6)	11 (25.0)	48 (33.8)	
E. coli	7 (7.9)	5 (11.4)	12 (8.5)	
Lactobacilli	40 (44.9)	28 (63.6)	68 (47.9)	
Streptococcus	5 (5.6)	0 (0.0)	5 (3.5)	
Bifidobacteria	37 (41.6)	11 (25.0)	48 (33.8)	
**Length of probiotics therapy for acute diarrhea (days)**				0.013
5	0 (0.0)	1 (2.1)	1 (0.7)	
7	8 (9.0)	7 (15.2)	15 (15.1)	
10	5 (5.6)	9 (19.5)	14 (10.4)	
14	19 (21.5)	13 (28.2)	32 (23.8)	
Until resolution of symptoms	13(14.7)	2 (4.3)	15 (11.1)	
Maintaining therapy for some days after resolution of symptoms	43 (48.8)	14 (30.3)	15 (11.1)	
**Use of probiotics to prevent antibiotic-associated diarrhea and C. difficile infection**				0.011
Yes	74 (85.0)	39 (67.2)	113 (77.9)	
No	13 (14.9)	19 (32.7)	32 (22.0)	
**Type of probiotic used to prevent antibiotic-associated diarrhea and C. difficile infection**				0.672
Multistrain formulations	58 (69.8)	26 (61.9)	84 (67.2)	
Bifidobacteria, Lactobacilli	1 (1.2)	0 (0.0)	1 (0.8)	
Single strain with Lactobacilli	8 (9.6)	2 (4.7)	10 (8.0)	
Single strain with Bifidobacteria	3 (3.6)	3 (7.1)	6 (4.8)	
Single strain with E. coli	9 (10.8)	8 (19.0)	17 (13.6)	
Single strain with Saccharomyces	1 (1.2)	1 (2.3)	2 (1.6)	
Other	3 (3.6)	2 (4.7)	5 (4.0)	
**Preferred bacterial genera in multistrain products for preventing antibiotic-associated diarrhea and C. difficile infection (multiple choice)**				0.018
Lactobacilli	19 (26.4)	19 (48.7)	38 (33.6)	

Bifidobacteria	23 (31.9)	11 (28.2)	34 (30.1)	
E. coli	0 (0.0)	2 (5.1)	2 (1.8)	
Saccharomyces	29 (40.3)	7 (17.9)	36 (31.9)	
Streptococcus	1 (1.4)	0 (0.0)	1 (0.9)	
**Prescription schedule for probiotics to prevent antibiotic-associated diarrhea and C. difficile infection.**				0.169
During and after antibiotic therapy	57 (67.8)	31 (73.8)	88 (69.8)	
Before starting antibiotic therapy	2 (2.3)	4 (9.5)	6 (4.7)	
Before and during antibiotic therapy	21 (25.0)	6 (14.2)	27 (21.4)	
Only during antibiotic therapy	4 (4.7)	1 (2.3)	5 (3.9)	
**Use of probiotics as adjuncts in the treatment of H. pylori eradication**				<0.001
Yes	79 (88.7)	29 (50.8)	108 (73.9)	
No	10 (11.2)	28 (49.1)	38 (26.0)	
**Use of probiotics in H. pylori eradication**				0.002
As a single eradication therapy	7 (8.3)	1 (2.7)	8 (6.6)	
Together with antibiotic therapy to exploit the effect of probiotics in inhibiting H. pylori growth through spatial competition, utilization of energy substrates, and production of bacteriocins	64 (76.1)	18 (50.0)	82 (68.3)	
None of the above reasons	3 (3.5)	6 (16.6)	9 (7.5)	
To reduce adverse events associated with antibiotic therapy to improve tolerability	10 (11.9)	11 (30.5)	21 (17.5)	
**Type of probiotic prescribed for H. pylori eradication**				0.349
Multistrain formulations	49 (61.2)	15 (48.3)	64 (57.6)	
Single strain with Bifidobacteria	2 (2.5)	2 (6.4)	4 (3.6)	
Single strain with E. coli	0 (0.0)	1 (3.2)	1 (0.9)	
Single strain with Lactobacilli	19 (23.7)	8 (25.8)	27 (24.3)	
Single strain with Saccharomyces	10 (12.5)	5 (16.1)	15 (13.5)	

Supplementary Table S3. Behavior of Gastroenterologists and Healthcare Professionals regarding the management of Irritable Bowel Syndrome with probiotics among participants in the survey.

	**Healthcare professionals n (%) *n* = 83**	**Gastroenterologist n (%) *n* = 59**	**Total n (%) *n* =142**	***p*-Value**
**Prescription schedule for probiotics in patients with IBS**				0.018
Probiotics with monthly cycles	65 (74.7)	44 (77.1)	109 (75.6)	
Continuous probiotics	16 (18.3)	3 (5.26)	19 (13.1)	
No	6 (6.9)	10 (17.5)	16 (11.1)	
**Type of probiotic prescribed for IBS**				0.043
Multistrain formulations	57 (71.2)	26 (55.3)	83 (65.3)	
Single strain with Lactobacilli	6 (7.5)	12 (25.5)	18 (14.1)	
Single strain with Bifidobacteria	15 (18.7)	6 (12.7)	21 (16.5)	
Single strain with E. coli	1 (1.2)	2 (4.2)	3 (2.3)	
Single strain with Saccharomyces	1 (1.2)	1 (2.1)	2 (1.5)	
**Preferred bacterial genera in multistrain products for IBS (multiple choice)**				0.108
Lactobacilli	24 (35.8)	19 (46.3)	43 (39.8)	
Bifidobacteria	33 (49.3)	14 (34.1)	47 (43.5)	
E. coli	0 (0.0)	3 (7.3)	3 (2.8)	
Saccharomyces	4 (6)	3 (7.3)	7 (6.5)	
Bacillus	6 (9)	2 (4.9)	8 (7.4)	
**Criteria for discontinuing continuous probiotic therapy in IBS**				<0.001
In case of inefficacy after 2 weeks	7 (8.7)	2 (5.5)	9 (7.7)	
In case of inefficacy after 4 weeks	7 (8.7)	7 (19.4)	14 (12.0)	
In case of inefficacy after 2 months	10 (12.5)	19(52.7)	29 (25.0)	
I do not discontinue therapy but change the type of probiotic	56 (70.0)	8 (22.2)	64 (55.1)	
**Duration of probiotics cycles for IBS (days)**				0.055
5	0 (0.0)	2 (4.1)	2 (1.6)	
7	8 (10.8)	10 (20.8)	18 (14.7)	
10	14 (18.9)	14 (29.1)	28 (22.9)	
14	33 (44.5)	14 (29.1)	47 (38.5)	
>14	19 (25.6)	8 (16.6)	27 (22.1)	
**Duration in months of probiotics cycles for IBS**				0.062
Mean (± SD)	3.8 (2.8)	4.4 (2.6)		

Supplementary Table S4. Behavior of Gastroenterologists and Healthcare Professionals regarding the management of Inflammatory Bowel Disease with probiotics among participants in the survey.

	Healthcare professionals n (%) *n* = 83	Gastroenterologist n (%) *n* = 59	Total n (%) *n* =142	*p*-Value
**Probiotics prescription for patients with Ulcerative Colitis**				<0.001
Yes, in patients in remission	7 (8.6)	17 (29.3)	24 (17.2)	
Yes, in patients with mild clinical activity	14 (17.2)	13 (22.4)	27 19.4)	
Yes, in patients with moderate clinical activity	19 (23.4)	17 (29.3)	36 (25.9)	
Yes, but only when I suspect an overlap with IBS	23 (28.4)	2 (3.4)	25 (17.9)	
I do not prescribe probiotics	18 (22.2)	9 (15.5)	27 (19.4)	
**Probiotics prescription for patients with Crohn's Disease**				<0.001
Yes, in patients in remission	20 (25.3)	10 (17.2)	30 (21.9)	
Yes, in patients with mild clinical activity	24 (30.3)	2 (3.4)	26 (18.9)	
Yes, in patients with moderate clinical activity	17 (21.5)	6 (10.3)	23 (16.7)	
Yes, but only when suspecting an overlap with IBS	12 (15.1)	14 (24.1)	26 (18.9)	
I do not prescribe probiotics	6 (7.5)	26 (44.8)	32 (23.3)	
**Type of probiotic prescribed for Ulcerative Colitis and Crohn's Disease**				0.022
Multistrain formulations	53 (76.8)	17 (56.6)	70 (70.7)	
Single strain with Lactobacilli	6 (8.7)	3 (10.0)	9 (9.0)	
Single strain with Bifidobacteria	7 (10.1)	2 (6.6)	9 (9.0)	
Single strain with E. coli	2 (2.9)	7 (23.3)	9 (9.0)	
Single strain with Saccharomyces	1 (1.4)	1 (3.3)	2 (2.0)	
**Criteria for discontinuing continuous probiotic therapy in Ulcerative Colitis**				0.002
In case of inefficacy after 2 weeks	8 (13.1)	4 (12.9)	12 (13.0)	
In case of inefficacy after 4 weeks	9 (14.7)	8 (25.8)	17 (18.4)	
In case of inefficacy after 2 months	9 (14.7)	12 (38.7)	21 (22.8)	
I do not discontinue therapy but change the type of probiotic	29 (47.5)	2 (6.4)	31 (33.7)	
**Duration of probiotics cycles for Ulcerative Colitis (days)**				0.048
5	2 (2.9)	0 (0.0)	2 (1.9%)	
7	5 (7.2)	4 (11.7)	9 (8.7%)	
10	13 (18.8)	15 (44.1)	28 (27.1%)	
14	34 (49.2)	11 (32.3)	45 (43.6%)	
>14	15 (21.7)	4 (11.7)	19 (18.4%)	
**Duration in months for probiotics cycles for Ulcerative Colitis**				0.634
Mean (± SD)	4.3 (3.1)	3.9 (2.8)		
**Criteria for suspending continuous probiotic therapy in Crohn's Disease**				0.000
In case of inefficacy after 2 weeks	9 (12.8)	3 (11.5)	12 (12.5)	
In case of inefficacy after 4 weeks	8 (11.4)	3 (11.5)	11 (11.4)	
In case of inefficacy after 2 months	9 (12.8)	15 (57.6)	24 (25.0)	
I do not suspend therapy but change the type of probiotic	35 (50.0)	1 (3.8)	36 (37.5)	
**Duration of probiotics cycles for Crohn's Disease (days)**				0.197
5	2 (2.9)	0 (0.0)	2 (2.1)	

7	5 (7.3)	3 (11.1)	8 (8.4)	
10	12 (17.6)	10 (37.0)	22 (23.1)	
12	1 (1.4)	0 (0.0)	1 (1.0)	
14	32 (47.0)	12 (44.4)	44 (46.3)	
>14	16 (23.5)	2 (7.4)	18 (18.9)	
**Duration in months of probiotics cycles for Crohn's Disease**				0.905
Mean (± SD)	4.1 (5.1)	4.2 (2.9)		

Supplementary Table S5. Behavior of Gastroenterologists and Healthcare Professionals regarding the management of Diverticular Disease with probiotics among participants in the survey.

	Healthcare professionals n (%) *n* = 83	Gastroenterologist n (%) *n* = 59	Total n (%) *n* =142	*p*-Value
**Probiotics prescription for patients with a history of diverticulosis**				0.001
Probiotics with monthly cycles	63 (77.7)	36 (62.0)	99 (71.2)	
Continuous probiotics	12 (14.8)	4 (6.9)	16 (11.5)	
No	6 (7.4)	18 (31.3)	24 (17.2)	
**Type of probiotic prescribed for patients with diverticulosis**				0.002
Multistrain formulations	41 (56.9)	18 (43.9)	59 (52.2)	
Single strain with Bifidobacteria	11 (15.2)	3 (7.3)	14 (12.3)	
Single strain with Lactobacilli	17 (23.6)	7 (17.0)	24 (21.2)	
Single strain with E. coli	3 (4.1)	11 (26.8)	14 (12.3)	
Single strain with Saccharomyces	0 (0.0)	2 (4.8)	2 (1.7)	
**Criteria for suspending continuous probiotic therapy in diverticulosis**				0.726
In case of inefficacy after 2 weeks	10 (15.6)	4 (13.3)	14 (14.8)	
In case of inefficacy after 4 weeks	12 (18.7)	6 (20.0)	18 (19.1)	
In case of inefficacy after 2 months	11 (17.1)	8 (26.6)	19 (20.2)	
I do not suspend therapy	31 (48.4)	12 (40.0)	43 (45.7)	
**Weeks before suspending probiotic therapy in diverticulosis**				0.804
Mean (± SD)	7.61 (7.76)	9.13 (7.74)		
**Duration of probiotics cycles for diverticulosis (days)**				0.008
5	1 (1.4)	2 (5.2)	3 (2.8)	
7	6 (8.7)	11 (28.9)	17 (15.8)	
10	15 (21.7)	12 (31.5)	27 (25.2)	
14	33 (47.8)	10 (26.3)	43 (40.1)	
>14	14 (20.2)	3 (7.8)	17 (15.8)	
**Duration in months of probiotics cycles for diverticulosis**				0.325
Mean (±SD)	3.7 (2.7)	4.4 (2.5)		
**Probiotics prescription for patients with SUDD**				0.209
Probiotics with monthly cycles	52 (69.3)	32 (57.1)	84 (64.1)	
Continuous probiotics	13 (17.3)	10 (17.8)	23 (17.5)	
No	1 (13.3)	14 (25.0)	24 (18.3)	
**Type of probiotic prescribed for SUDD**				0.165
Multistrain formulations	37 (58.7)	21 (51.2)	58 (55.7)	
Single strain with Lactobacilli	13 (20.6)	8 (19.5)	21 (20.1)	
Single strain with Bifidobacteria	8 (12.7)	3 (7.3)	11 (10.5)	
Single strain with E. coli	2 (3.1)	7 (17.0)	9 (8.6)	
Single strain with Saccharomyces	3 (4.7)	2 (4.8)	5 (4.8)	
**Criteria for suspending continuous probiotic therapy in SUDD**				0.237
In case of inefficacy after 2 weeks	11 (20.7)	7 (20.5)	18 (20.6)	
In case of inefficacy after 4 weeks	9 (16.9)	6 (17.6)	15 (17.2)	
In case of inefficacy after 2 months	11 (20.7)	13 (38.2)	24 (27.5)	

I do not suspend therapy but change the type of probiotic	22 (41.5)	8 (23.5)	30 (34.4)	
**Duration of probiotics cycles for SUDD (days)**				0.013
5	2 (3.4)	2 (5.5)	4 (4.2)	
7	3 (5.1)	11 (30.5)	14 (14.8)	
10	14 (24.1)	8 (22.2)	22 (23.4)	
14	28 (48.2)	12 (33.3)	40 (42.5)	
>14	11 (18.9)	3 (8.3)	14 (14.8)	
**Duration in months of probiotics cycles for SUDD**				0.167
Mean (± SD)	4.9 (3.7)	5.6 (3.3)		
**Probiotics prescription for patients with a history of acute diverticulitis**				0.002
Probiotics with monthly cycles	43 (63.2)	31 (56.3)	74 (60.1)	
Continuous probiotics	9 (13.2)	20 (36.3)	29 (23.5)	
No	9 (13.2)	20 (36.3)	29 (23.5)	
**Type of probiotic prescribed for patients with a history of acute diverticulitis**				0.053
Multistrain formulations	37 (61.6)	17 (44.7)	54 (55.1)	
Single strain with Lactobacilli	9 (15.0)	3 (7.8)	12 (12.2)	
Single strain with Bifidobacteria	3 (5.0)	9 (23.6)	12 (12.2)	
Single strain with E. coli	9 (15.0)	8 (21.0)	17 (17.3)	
Single strain with Saccharomyces	2 (3.3)	1 (2.6)	3 (3.0)	
**Criteria for discontinuing continuous probiotic therapy in patients with a history of acute diverticulitis**				0.226
In case of inefficacy after 2 weeks	10 (18.1)	5 (16.1)	15 (17.4)	
In case of inefficacy after 4 weeks	10 (18.1)	6 (19.3)	16 (18.6)	
In case of inefficacy after 2 months	11 (20.0)	12 (38.7)	23 (26.7)	
I do not discontinue the therapy but change the type of probiotic	24 (43.4)	8 (25.8)	32 (37.2)	
**Duration in months for continuous probiotic therapy in patients with a history of acute diverticulitis**				0.025
Mean (± SD)	4.3 (3.5)	5.7 (3.4)		
**Duration of probiotics cycles for patients with a history of acute diverticulitis (days)**				0.047
5	3 (5.2)	0 (0.0)	3 (3.3)	
7	4 (7.0)	10 (29.4)	14 (15.3)	
10	13 (22.8)	7 (20.5)	20 (21.9)	
14	27 (47.3)	13 (38.2)	40 (43.9)	
>14	10 (17.5)	4 (11.7)	14 (15.3)	
**Duration in months of probiotics cycles for acute diverticulitis**				0.025
Mean (± SD)	4.3 (3.5)	5.7 (3.4)		
